# Opportunistic Detection of Chronic Kidney Disease Using CT-Based Measurements of Kidney Volume and Perirenal Fat

**DOI:** 10.3390/jcm14165888

**Published:** 2025-08-20

**Authors:** Piotr Białek, Michał Żuberek, Adam Dobek, Krzysztof Falenta, Ilona Kurnatowska, Ludomir Stefańczyk

**Affiliations:** 1 1st Department of Radiology and Diagnostic Imaging, Medical University of Lodz, Kopcinskiego 22 Street, 90-153 Lodz, Poland; 2 Department of Internal Diseases and Transplant Nephrology, Medical University of Lodz, Kopcinskiego 22 Street, 90-153 Lodz, Poland

**Keywords:** opportunistic screening, chronic kidney disease, kidney volume, perirenal fat, renal hilum fat, renal hilum attenuation, computed tomography, abdominal fat, visceral fat, imaging biomarkers

## Abstract

**Background/Objectives:** Chronic kidney disease (CKD) is a prevalent condition with many cases remaining undiagnosed, although early detection is essential. Adipose tissue distribution—particularly perirenal fat thickness (PrFT)—has recently been linked to renal pathophysiology. This study assessed the association between CT-derived parameters of fat distribution and kidney morphology with CKD. **Materials and Methods:** This retrospective study included 237 patients (117 subjects, 120 controls) who underwent abdominal CT and had serum creatinine data. The dataset was randomly split (70% training, 30% test) to develop and evaluate a logistic regression model. CKD was defined as estimated Glomerular Filtration Rate (eGFR) < 60 mL/min/1.73 m^2^. PrFT was measured as the distance from the posterior renal capsule to the posterior abdominal wall; renal hilum fat was segmented using a −195 to −45 HU range. Additional parameters (measured using automated segmentation tools) included kidney volume (KV), visceral/subcutaneous fat areas, skeletal muscle area and attenuation, and liver attenuation. Bilateral measurements were averaged. **Results:** KV (OR = 0.249, 95% CI: 0.146–0.422, *p* < 0.001) and PrFT (2nd tercile: OR = 7.720, 95% CI: 2.860–20.839; 3rd tercile: OR = 16.892, 95% CI: 5.727–49.822; both *p* < 0.001) were identified as independent predictors of CKD. These variables were used to construct a simplified model, which demonstrated moderate clinical applicability (AUC = 0.894) when evaluated on the test subset. **Conclusions:** KV and PrFT emerged as independent predictors of CKD, forming the basis of a simplified model with potential for opportunistic clinical application. This approach may facilitate earlier detection of CKD in patients undergoing CT imaging for unrelated clinical reasons. These imaging parameters are not intended to replace serum creatinine or eGFR but may serve as complementary predictors in specific clinical contexts.

## 1. Background

Chronic kidney disease (CKD) is a prevalent (affecting around 10% of population) [1,2,3,4,5] and progressive [5,6] condition. CKD is typically screened for and staged using serum creatinine levels and estimated Glomerular Filtration Rate (eGFR); while measured GFR would be more accurate for diagnosis, it is rarely feasible in clinical practice, making eGFR the standard tool in most settings [2,5]. It is important to emphasize that the kidneys possess a substantial functional reserve, and before excretory dysfunction manifested by a decline in eGFR becomes apparent, certain morphological markers indicative of renal injury may already be detectable, such as kidney volume (KV). Many patients remain unaware of their condition and that early stages of CKD are often asymptomatic. Timely medical interventions can slow its progression, so finding strategies of early detection has become increasingly important [4,7]. In recent years, imaging has gained a complementary role in nephrology, providing valuable insights into renal morphology and the surrounding tissue environment [8,9]. Among emerging risk factors, the distribution of adipose tissue has drawn increasing attention due to its potential local and systemic effects on kidney function [1,10].

While visceral fat is well recognized for its association with metabolic dysfunction—contributing to insulin resistance [11,12] and systemic inflammation marked by interleukin-6 (IL-6), tumor necrosis factor-α (TNF-α), and macrophage chemoattractant protein-1 (MCP-1) [11,12]—growing evidence [13] suggests that local fat depots, such as perirenal fat and renal hilum fat, may play a more direct role in renal pathophysiology [13,14,15]. Perirenal fat, located between the kidney and renal fascia [15,16], may influence CKD progression through mechanical compression [16,17,18] as well as endocrine (adiponectin, leptin, visfatin, resistin) [13] and inflammatory signaling (IL-6, TNF- α and MCP-1) [13,14,15,17,18], while hilum fat, surrounding the renal vessels, may compress low-pressure renal venous structures affecting renal perfusion and hemodynamics [17,19].

Previous studies have largely examined associations between perirenal fat thickness (PrFT) and CKD in patients with type 2 diabetes [18,20,21] or cardiovascular diseases [16,18,22], where abnormal fat distribution and CKD risk are both elevated. However, a recent meta-analysis by Kang et al. [18] suggests that the relationship between perirenal fat and CKD may extend beyond diabetic patients, indicating a more generalizable association across different populations. Despite this, the field remains underexplored, particularly in non-diabetic cohorts, and the precise role of regional fat compartments in CKD pathogenesis is yet to be fully clarified.

In addition to fat distribution, KV may play a role as an imaging marker that reflects parenchymal mass and structural remodeling [9,23,24,25,26,27,28,29]. Decreased KV has been consistently associated with reduced nephron number and declining renal function [23]. While KV directly reflects anatomical changes related to disease severity [27], fat-based markers may offer earlier or complementary insights into CKD pathophysiology through their metabolic and mechanical effects.

This study aims to evaluate the relationship between CT-derived measurements of KV, PrFT, renal hilum volume, renal hilum attenuation, visceral fat area, subcutaneous fat area, skeletal muscle area, skeletal muscle attenuation, and liver steatosis with the presence of CKD. By assessing both local and systemic adipose compartments alongside renal morphology, we aim to clarify their relative contributions to renal impairment and explore their potential as opportunistic imaging biomarkers for early detection and risk stratification of CKD.

## 2. Materials and Methods

### 2.1. Ethics

This retrospective case-control study was conducted in accordance with the Declaration of Helsinki and received approval from the local Bioethics Committee (Approval No. RNN/174/24/KE; approval date: 9 July 2024). Informed consent for participation was obtained from all subjects involved in the study.

### 2.2. Inclusion and Exclusion Criteria

The study comprised patients who underwent abdominal computed tomography (CT) scans performed between July 2019 and July 2024 which were retrospectively analyzed. All scans were acquired using a GE Revolution CT 64-slice scanner (GE Healthcare, Chicago, IL, USA) with acquisition parameters of 120 kV and 2.5 mm slice thickness. Although most examinations included multiphasic contrast-enhanced phases, only non-contrast (pre-contrast) images were used for this analysis, and all scans were evaluated exclusively in the non-contrast phase to ensure methodological consistency and enable reliable analysis and comparison of results. Non-contrast phases were more available, as multiphasic studies often include a non-contrast phase. Importantly, contrast enhancement is not expected to affect KV or the appearance of normal adipose tissue, which does not enhance after contrast administration. Therefore, based on our methodology, we consider non-contrast images sufficient and appropriate for model development, and we anticipate that the model will be generalizable to both non-contrast and contrast-enhanced studies.

Our institutional image database was screened for patients with a formal referral with an explicitly stated diagnosis of CKD, typically from a nephrologist, internist, or primary care physician. The fact that the patient was explicitly referred as having CKD was taken as confirmation that the chronicity criterion (>3 months) had been met. Additionally, two further inclusion criteria had to be met: (1) a serum creatinine result obtained within 14 days prior to the CT scan, in accordance with institutional policy; and (2) an eGFR < 60 mL/min/1.73 m^2^, calculated using the MDRD (Modification of Diet in Renal Disease) formula, corresponding to CKD stages III, IV, and V according to the Kidney Disease: Improving Global Outcomes (KDIGO) classification. Only patients meeting all these criteria were included [30].

A numerically matched control group was selected, consisting of individuals with no known history of kidney disease, an eGFR ≥ 60 mL/min/1.73 m^2^, and a serum creatinine result available within 14 days prior to a CT scan performed during the same time period for non-renal indications. While this 1:1 distribution does not reflect the actual prevalence of CKD [1,2,3,4,5], it was intentionally chosen to evaluate the discriminative performance of our model. No statistical weighting was applied.

The same exclusion criteria were applied to both groups. Exclusion criteria included

1. Acute kidney injury at the time of imaging;
2. Extensive free fluid or fat stranding impairing fat quantification (determined during screening by researchers to avoid visceral fat tissue misclassification due to abnormal density);
3. Renal transplants;
4. Hydronephrosis;
5. Renal neoplasms (simple renal cysts were not an exclusion criterion);
6. Polycystic kidney disease;
7. Significant image artifacts.

### 2.3. Image Analysis

Further image analysis was performed using 3D Slicer software (version 5.8.1, www.slicer.org) with the TotalSegmentator extension (version 8426cdf, GitHub) [31]. This extension enabled

**Figure 1:**
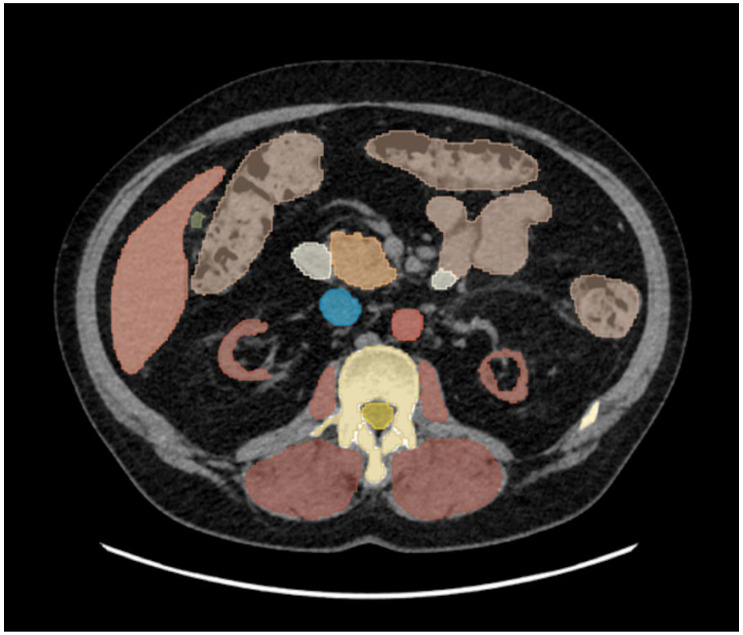
Automated all organ segmentation using the TotalSegmentator extension in 3D Slicer software in a patient with stage V chronic kidney disease. The kidney parenchyma is segmented and shown in red bilaterally, with the renal hilum not included. Segmentation of the remaining organs, shown in various colors, is presented only to illustrate the software output.

- Automated segmentation of all major abdominal organs for assessment of KV (Figure 1) (including liver and spleen attenuation). Only the kidney parenchyma was segmented with exclusion of renal hilum or cystic areas in subjects with renal cysts.

**Figure 2:**
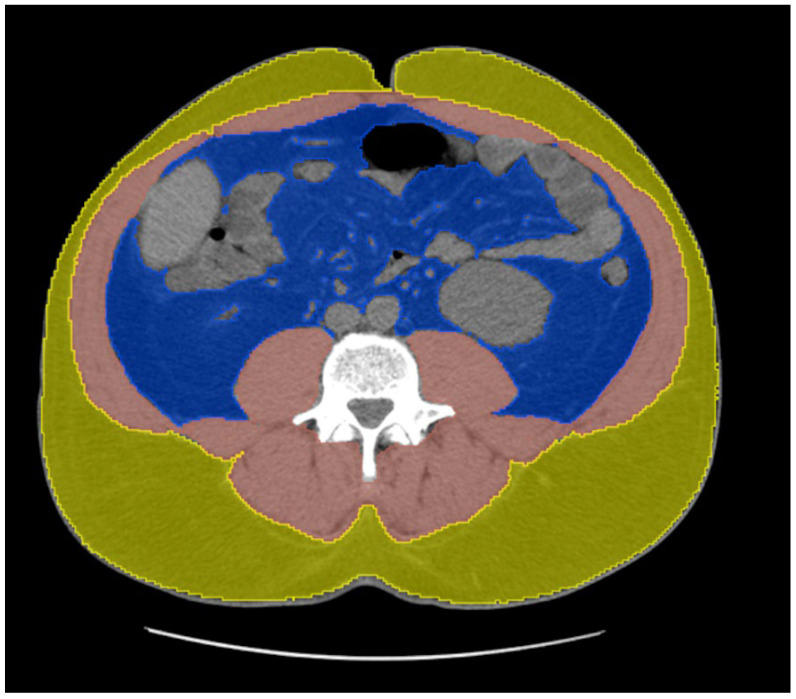
Automated tissue-type segmentation in 3D Slicer using the TotalSegmentator extension at the level of the inferior endplate of the L3 vertebra. Visceral fat is shown in blue, subcutaneous fat in yellow, and skeletal muscle in red.

Tissue-type segmentation was performed at the level of the lower endplate of the L3 vertebra [32] (Figure 2) using TotalSegmentator [31] AI algorithm to quantify four body composition parameters: visceral fat area, subcutaneous fat area, skeletal muscle area, and skeletal muscle attenuation. Visceral fat area was defined as the cross-sectional area of adipose tissue located within the abdominal cavity, surrounding internal organs and enclosed by the peritoneum. Subcutaneous fat area referred to the adipose tissue located between the skin and the outer border of the abdominal wall musculature. Skeletal muscle area represented the total cross-sectional area of the skeletal muscles visible at the L3 level, including the psoas, paraspinal, and abdominal wall muscles. Skeletal muscle attenuation was defined as the mean radiodensity of these muscles, measured in Hounsfield units [HU].

**Figure 3:**
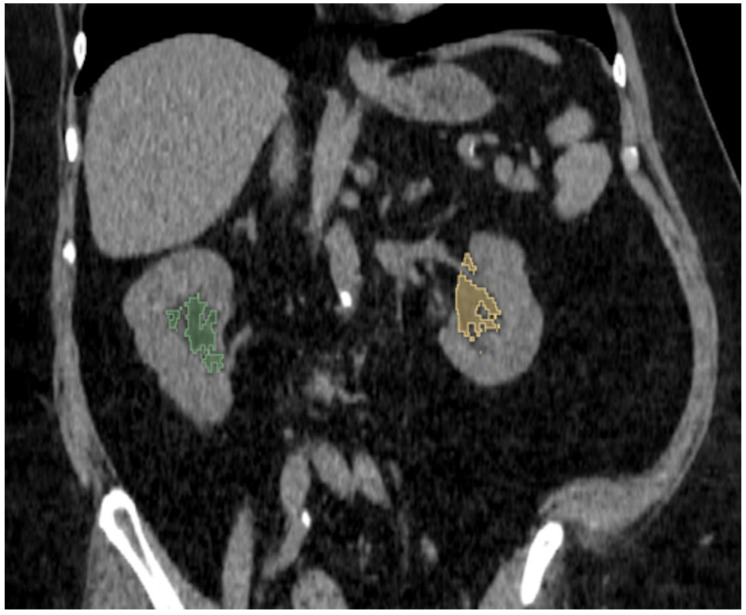
Manual segmentation of renal hilum fat is presented in coronal plane. The green area represents the right renal hilum, and the yellow area represents the left renal hilum. Fat was segmented using a Hounsfield unit threshold range of −195 to −45.

Fat in the renal hilum was segmented using a semi-automated approach based on HU thresholding within the range of −195 to −45 HU (Figure 3) [33]. For the purpose of this analysis, the entire fat content within the central renal region (renal sinus) was segmented and referred to as renal hilum fat. The operator manually indicated the approximate location of the fat using a mouse cursor, but the final segmentation was determined solely by voxel density values within the specified HU range. As a result, even if the region was incorrectly clicked (e.g., on renal parenchyma or vasculature), those structures were automatically excluded from the segmentation due to their attenuation values falling outside the predefined threshold.

Hepatic steatosis was defined as present when liver attenuation was >10 HU lower than spleen attenuation [34] or liver attenuation was lower than 40 HU in asplenic participants [35].

**Figure 4:**
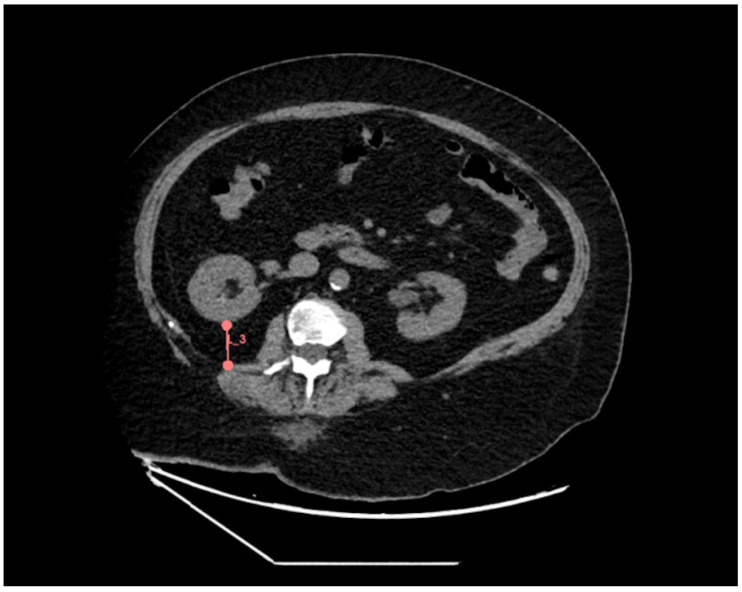
Illustration of posterior perirenal fat thickness measurement. The red line indicates the distance between the posterior renal capsule and the posterior abdominal wall at the level of the renal vein.

Posterior PrFT was measured at the level of the renal vein as the direct distance from the posterior renal capsule to the posterior abdominal wall (Figure 4) [16,32,36,37,38,39,40]. To ensure consistency and comparability with previously published studies [16,32,36,37,38,39,40], we adopted this commonly used definition, despite its anatomical imprecision, specifically, the inclusion of pararenal fat. This approach remains widely accepted in CT-based research [16,32,36,37,38,39,40] (and often in ultra-sound- and MRI-based studies) [17,41], likely because the renal fascia is frequently poorly visualized on CT. Therefore, it also allowed us to avoid excluding a substantial number of patients due to limited fascia visibility in our cohort.

Measurements including KV, renal hilum volume, renal hilum attenuation, and PrFT were performed bilaterally, and the mean of both kidneys was used for analysis.

All volumetric, surface area, and attenuation measurements were obtained using the built-in tools in 3D Slicer. All segmentations were independently reviewed and approved by two radiologists by consensus. In cases of discrepancy, a third radiologist with over 30 years of experience in abdominal radiology was consulted to resolve the issue. All of them were blinded to participants diagnoses.

**Figure 5:**
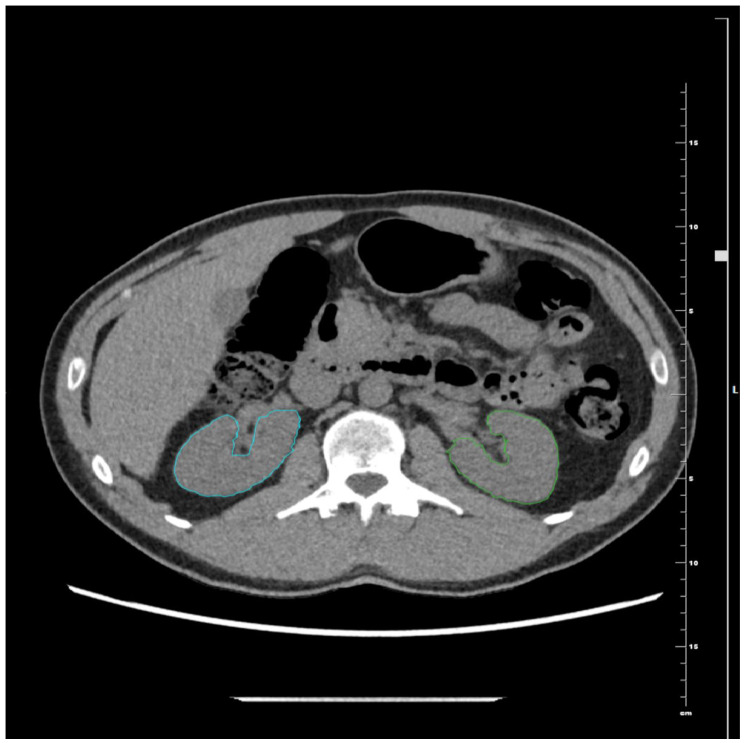
Kidneys segmentation performed in Exhibeon software using the 3D Smart Brush tool in healthy control. Only the kidney parenchyma was segmented. The renal hilum is not included in the segmentation. Blue contour represents right kidney, and green contour represents left kidney.

To assess reproducibility, a randomly selected and equally sized subset of participants from both the study and control groups was used. The same predefined panel of radiologists, still blinded to clinical data, repeated renal hilum measurements after a three-month interval using the same software (3D Slicer). Given the anticipated clinical importance of KV and PrFT, these two parameters were also reassessed after three months but using a different platform: Exhibeon version 4.8.0 (Pixel Technology, Łódź, Poland). KV was measured using semi-automated 3D Smart Brush segmentation tool (Figure 5), while PrFT was measured manually in the same manner as originally. All repeat measurements were performed by the same radiologist group under consensus conditions. Reproducibility of KV, PrFT, and measurements of renal hilum parameters were evaluated using the intraclass correlation coefficient (ICC 3.1, two-way mixed-effects model, absolute agreement). In addition, paired *t*-tests were conducted to assess whether there were any significant differences between the initial and repeat measurements. Agreement between measurements was also examined using the Bland–Altman analysis, including calculation of limits of agreement (LoA).

### 2.4. Statistical Analysis

The Shapiro–Wilk test was used to assess the normality of data distribution. Based on its results, continuous variables with normal distribution are presented as mean ± standard deviation (SD), while those with non-normal distribution are reported as median and interquartile range (IQR). Categorical variables are presented as counts and percentages.

For group comparisons, the independent samples Student’s *t*-test was applied to continuous variables with normal distribution, and the Mann–Whitney U test was used for those not normally distributed; both tests were two-tailed. Categorical variables were compared using the chi-square test of independence.

The dataset was randomly divided into a training set (70%) and a test set (30%) using stratified sampling to ensure balanced distribution of the dependent variable. All modeling and variable selection steps were performed on the training set.

Univariate logistic regression (*p* < 0.10) was used to identify candidate predictors. The Box–Tidwell test was then applied to assess the linearity assumption for continuous variables. Continuous predictors were Z-standardized, and candidate predictors were entered into a multivariate logistic regression model.

Subsequently, the multivariate model was simplified within the training set by retaining only statistically significant predictors (*p* < 0.05) Influential observations were identified using Cook’s distance (threshold: 4/*n*) and were further examined for potential data errors. Multicollinearity was assessed using the Variance Inflation Factor (VIF) and tolerance, with thresholds of VIF > 5 or tolerance < 0.2 indicating potential issues. Sample size adequacy was assessed using the Events Per Variable (EPV) ratio, with a recommended threshold of ≥10.

Model fit was evaluated in the training set using the Nagelkerke pseudo-R^2^. The final logistic regression model, developed on the training set, was then tested on the independent test set. Its predictive performance was assessed using the area under the receiver operating characteristic curve (ROC AUC), and the resulting AUCs from both sets were compared.

Statistical analysis was performed using JASP software (version 0.19.3). Observations with missing data were handled by omission. A *p*-value < 0.05 was considered statistically significant.

## 3. Results

The study included 237 participants: 117 patients with impaired kidney function and 120 control subjects with normal kidney function. A summary of the cohort’s characteristics is presented in Table 1.

A stratified random sample of 30 participants per group demonstrated consistently high reproducibility of measurements (Table 2).

The training and test sets consisted of 166 and 71 observations, respectively.

Univariate logistic regression analysis (Table 3) identified age, KV, PrFT, and skeletal muscles attenuation as candidate predictors, which were subsequently included in the multivariate logistic regression model.

PrFT violated the Box–Tidwell assumption (*p* < 0.001) and was therefore categorized into terciles. Other predictors met the linearity in logit assumption (*p* > 0.05).

Results from the multivariate logistic regression are shown in Table 4. Only KV and PrFT emerged as independent predictors of CKD. The model was simplified to include only significant predictors (KV and PrFT; Table 5). Since the AUC remained unchanged, we selected the simplified model, although the explanatory power slightly decreased. Nagelkerke R^2^ was 0.493. Therefore, the simplified model was preferred due to its comparable performance and greater parsimony. EPV was 27.7. No multicollinearity issues occurred (VIF < 5, tolerance > 0.2). Eleven observations with Cook’s distance > 4/*n* were reviewed but retained in the analysis.

**Figure 6:**
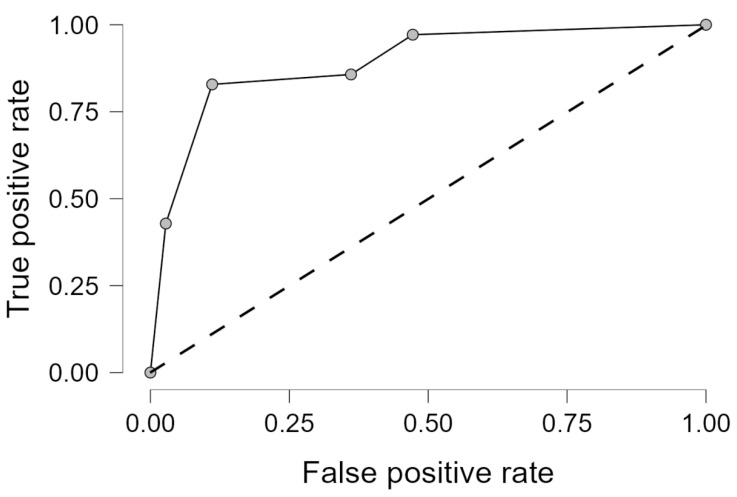
Receiver operating characteristic (ROC) curve of the simplified multivariable logistic regression model on the test dataset (AUC = 0.894).

Upon evaluation of the test dataset, the AUC increased from 0.874 to 0.894. Additional performance metrics are provided in Table 6, and the ROC curve of the test model is presented in Figure 6.

## 4. Discussion

In this imaging-based analysis, KV and PrFT, both measured on unenhanced CT images, were identified as independent predictors of CKD. The simplified model including only KV and PrFT explained a substantial portion of the variance in CKD status (pseudo-Nagelkerke R^2^ = 0.493), indicating that these two imaging features have strong explanatory power in this setting. The AUC even increased in the test set, reaching 0.894, indicating strong discriminative performance.

Consistent with the prior imaging literature [23,24,25,26,27,28,29,30,31,32,33,34,35,36,37,38,42], KV was inversely associated with CKD, reflecting structural remodeling and nephron loss in chronic disease states [24]. This is further supported by Gupta et al. [27], who reported a moderate correlation between renal volume measured on unenhanced CT and eGFR (r = 0.65, *p* < 0.001), along with a strong correlation with split renal function estimates from radionuclide renography (r = 0.95, *p* < 0.001), reinforcing the value of renal volumetry as a possible early indicator of renal impairment prior to measurable decline in eGFR. Similarly, Choi et al. [23] and Goh et al. [25] compared volume-based GFR estimates derived from CT with direct GFR measurements and found that their newly developed volume-based formula outperformed traditional creatinine-based methods in accuracy in healthy kidney donors.

PrFT emerged as an independent risk factor for CKD, although its effect was nonlinear. The strength of this association was reflected in high odds ratios observed in both univariate and multivariate analyses. This supports prior evidence that perirenal fat may directly contribute to renal function. Previous studies have shown its relevance mostly in diabetic populations. For instance, Fang et al. [43], using ultrasound-based measurements of PrFT, demonstrated a significant negative correlation between PrFT and eGFR in patients with type 2 diabetes, particularly in men, even after adjusting for confounding factors. Similarly, Lamacchia et al. [20] found that both para- and PrFT thickness measured on ultrasound were independent predictors of kidney dysfunction, outperforming waist circumference and body mass index (BMI) in predicting lower eGFR, renal resistance index, and uricaemia. Beyond metabolic and diabetic populations, PrFT has also been implicated in cardiorenal interactions. Cho et al. [16] investigated patients hospitalized with acute decompensated heart failure and demonstrated that increased posterior PrFT measured on CT was independently associated with reduced kidney function.

Building on the rationale proposed by Kang et al. [18], we aimed to develop a broadly applicable model for opportunistic CKD detection that does not rely on prior knowledge of disease etiology. For this reason, we did not stratify our analysis according to specific causes of CKD. While our cohort included patients with known comorbidities such as diabetes, hypertension, and glomerulonephritis—with the latter with histopathologically confirmation—etiology-specific analysis was beyond the scope of this work. Importantly, although the model was evaluated in a deliberately balanced dataset (1:1 CKD vs. controls), our intention was to investigate its general discriminative ability across etiologically diverse cases rather than to reflect true disease prevalence.

It should also be noted that referral based on confirmed CKD status was one of the inclusion criteria, which led to a heavily GFR-biased CKD group, skewed toward more advanced stages of the disease. This bias reflects the design of the study rather than a sampling error, as individuals with established CKD were purposefully selected for inclusion in that group. While our inclusion criteria allowed for CKD stages III–V, stage V accounted for 19 of 117 subjects in the CKD group (16.2%) and 19 of 237 participants (8.0%) overall, with the majority of CKD patients being in stages III–IV.

Notably, in our study, visceral fat area was not a statistically significant predictor of CKD. This unexpected finding contrasts with previous reports by Qin et al. [1] and Manabe et al. [10], who identified a positive relationship between visceral adiposity and decreased renal function. The observed discrepancy may be explained by characteristics specific to our cohort. The positive skewness of 1.036 suggests a concentration of lower values with a tail of higher measurements, which may have affected the overall distribution and outcome. Therefore, the role of visceral fat area in CKD should be interpreted with caution, and further investigation in larger, more diverse cohorts is warranted to clarify this relationship.

Our finding that liver steatosis was not significantly associated with CKD contrasts with previous studies and meta-analyses, which have reported a link between non-alcoholic fatty liver disease (NAFLD) and increased CKD risk [44,45]. For instance, a large meta-analysis by Musso et al. [44]—which included 33 studies and over 63,000 participants—demonstrated that both the presence and severity of NAFLD are associated with an increased risk and severity of CKD. Meta-analysis by Mantovani et al. [45] reported that NAFLD is associated with a nearly 40% increased risk of incident CKD. The discrepancy in our findings may be due to the limited sample size or differences in patient characteristics.

Importantly, most of the input parameters were derived through fully or semi-automated methods (requiring only region selection). Only PrFT required fully manual measurement. This level of automation supports the feasibility of developing an opportunistic artificial intelligence-based screening tool for CKD. Such a model could automatically raise suspicion for CKD in individuals undergoing abdominal CT scans performed for unrelated clinical reasons. For example, a patient presenting to the emergency department with non-specific abdominal pain may undergo a CT scan to exclude acute pathology; however, this same scan could also be leveraged to opportunistically raise suspicion for previously unrecognized CKD. Conversely, in cases where laboratory tests reveal elevated serum creatinine but the CT scan lacks imaging features typically associated with CKD, the model may suggest an acute kidney injury rather than chronic process. Given the high prevalence of undiagnosed CKD and the asymptomatic nature of its early stages [4,7], opportunistic detection using existing imaging data may provide a valuable pathway for earlier diagnosis and intervention. Further research is needed to validate this concept.

As this study focused on developing a simplified model for opportunistic CKD detection, we limited our analysis to absolute imaging parameters. Although we considered evaluating the ratio of PrFT to visceral or subcutaneous fat area, such proportional measures were beyond the intended scope. However, previous studies, such as that by Kawasaki et al. [46], have demonstrated a strong correlation between PrFT visceral fat area, supporting the physiological relevance of such comparisons. The use of proportional fat indices may therefore represent an interesting direction for future research.

This study has several limitations. It was a single-center retrospective study without external validation, which may limit the generalizability of the findings. Owing to its retrospective and cross-sectional nature, it did not include prospective follow-up to assess the association of imaging findings with CKD progression rate, which would be valuable for clinical application. Such longitudinal evaluation, although beyond the scope of our design, should be addressed in future studies. The modest cohort size may also limit the statistical power of the analysis and warrants validation in larger, independent populations. CKD etiologies associated with normal or enlarged KV, such as diabetic nephropathy or severe nephrotic syndrome, were included by design, as this was a deliberate choice consistent with the study’s aim of developing a broadly applicable model across diverse CKD etiologies. CKD status was based on referral from the attending physician and served as the basis for group allocation, and although only a single serum creatinine and eGFR value was available, this did not affect patient classification, which relied on clinical confirmation rather than laboratory thresholds alone. Most clinical data were available, though some variables had missing values and were handled by omission when necessary, particularly BMI, which could not be reliably calculated in several cases due to missing height data. Laboratory data relevant to kidney image findings beyond serum creatinine and eGFR were not consistently available and therefore were not analyzed; their inclusion is valuable for future studies, while their absence here also maintained the simplicity of the model. While most imaging parameters were obtained using automated or semi-automated methods, PrFT was measured manually; to address this, segmentations were performed and evaluated by the same group of radiologists, with reproducibility assessed after 3 months.

## 5. Conclusions

KV and PrFT emerged as independent predictors of CKD, forming the basis of a simplified model with potential for opportunistic clinical application. This approach may facilitate earlier detection of CKD in patients undergoing CT imaging for unrelated clinical reasons. These imaging parameters are not intended to replace serum creatinine or eGFR but may serve as complementary predictors in specific clinical contexts.

## Figures and Tables

**Table 1:** Study cohort characteristics.

	CKD	Controls	*p*-Value
Number	117 (100%)	120 (100%)	-
Male (*n*)	56 (47.9%)	61 (50.8%)	0.648
Age (years)	72.0 (60.0–78.0)	67.5 (49.0–74.3)	0.017
BMI (kg/m^2^)	25.4(±5.0)	26.4(±3.8)	0.114
Creatinine (mg/dL)	2.00 (1.45–3.11)	0.73 (0.62–0.85)	<0.001
eGFR (mL/min/1.73 m^2^)	29.0 (18.7–39.3)	96.6 (77.0–114.9)	<0.001
KV (mL)	106.1 (73.3–143.6)	154.2 (127.5–188.7)	<0.001
PrFT (mm)	15.12 (12.20–22.87)	8.27 (5.53–13.53)	<0.001
Renal hilum volume (mL)	8.41 (5.57–18.77)	6.88 (4.20–12.21)	0.007
Renal hilum attenuation (HU)	−70.0 (±9.4)	−66.7(±10.5)	0.012
Visceral fat area (cm^2^)	156.54 (91.96–236.67)	126.24 (66.68–197.91)	0.013
Subcutaneous fat area (cm^2^)	185.49 (126.34–266.54)	170.50 (118.92–255.28)	0.485
Skeletal muscles area (cm^2^)	112.56 (96.05–130.53)	115.58 (97.95–138.01)	0.421
Skeletal muscles attenuation (HU)	23.60 (15.40–32.10)	26.65 (17.48–34.80)	0.042
Liver steatosis (*n*)	13 (11.1%)	21 (17.5%)	0.161
Liver attenuation (HU)	54.3 (50.2–58.7)	51.4 (46.6–56.0)	<0.001

Normally distributed data are presented as mean ± standard deviation (SD), while non normally distributed data are presented as median with quartile range (Q1–Q3); eGFR—estimated glomerular filtration rate; KV—kidney volume; PrFT—perirenal fat thickness; HU—Hounsfield units.

**Table 2:** Reproducibility of quantitative measurements (*n* = 60).

	ICC (3,1) (95% CI)	Paired *t*-Test-*p*-Value	Bland–Altman (Mean Diff., LoA, Range)
KV	0.999 (0.998–0.999)	*p* = 0.546	0.184; −4.416 to 4.784; range: 9.200
PrFT	0.996 (0.993–0.998)	*p* = 0.299	−0.108; −1.675 to 1.459; range: 3.134
Renal hilum volume	0.997 (0.995–0.998)	*p* = 0.057	−0.129; −1.140 to 0.881; range: 2.021
Renal hilum attenuation	0.997 (0.995–0.998)	*p* = 0.135	0.135; −1.217 to 1.487; range: 2.704

KV—kidney volume; PrFT—perirenal fat thickness; ICC—intraclass correlation coefficient; CI—confidence interval; LoA—limits of agreement.

**Table 3:** Univariate logistic regression analysis of variables.

Predictor	OR (95% CI)	*p*-Value
Gender	0.616 (0.334–1.137)	0.121
Age	1.018 (1.001–1.035)	0.043
BMI	0.931 (0.852–1.018)	0.117
KV	0.978 (0.970–0.986)	<0.001
PrFT	1.125 (1.071–1.182)	<0.001
Renal hilum volume	1.034 (0.994–1.076)	0.103
Renal hilum attenuation	0.982 (0.953–1.012)	0.238
Visceral fat area	1.020 (0.999–1.005)	0.166
Subcutaneous fat area	1.000 (0.998–1.003)	0.797
Skeletal muscles area	0.993 (0.983–1.003)	0.148
Skeletal muscles attenuation	0.973 (0.950–0.997)	0.026
Liver steatosis	0.694 (0.289–1.667)	0.414
Liver attenuation	1.065 (1.021–1.111)	0.138

OR—odds ratio; CI—confidence interval; BMI—body mass index; KV—kidney volume; PrFT—perirenal fat thickness.

**Table 4:** Multivariate logistic regression model constructed on training dataset.

Variable	OR	OR Lower 95% CI	OR Upper 95% CI	*p*-Value
Intercept	0.173	0.077	0.389	<0.001
Age	0.732	0.410	1.307	0.292
KV	0.241	0.139	0.416	<0.001
PrFT (2nd tertile)	8.189	2.916	22.994	<0.001
PrFT (3rd tertile)	19.129	6.012	61.993	<0.001
Skeletal muscles attenuation	0.833	0.480	1.445	0.516

AUC 0.874; Nagelkerke pseudo-R^2^ 0.498. OR—odds ratio; CI—confidence interval; GFR—estimated glomerular filtration rate; AUC—area under the curve; KV—kidney volume; PrFT—perirenal fat thickness.

**Table 5:** Simplified multivariate logistic regression model constructed on training dataset.

Variable	OR	OR Lower 95% CI	OR Upper 95% CI	*p*-Value
Intercept	0.180	0.083	0.392	<0.001
KV	0.249	0.146	0.422	<0.001
PrFT (2nd tertile)	7.720	2.860	20.839	<0.001
PrFT (3rd tertile)	16.892	5.727	49.822	<0.001

AUC 0.874; Nagelkerke pseudo-R^2^ 0.493; OR—odds ratio; CI—confidence interval; AUC—area under the curve; KV—kidney volume; PrFT—perirenal fat thickness.

**Table 6:** Performance metrics of the simplified multivariable logistic regression model on the test dataset.

	Average/Total
***n***	71
AUC	0.894
Accuracy	0.789
F1 Score	0.800
Precision (Positive Predictive Value)	0.750
Recall (True Positive Rate (Sensitivity).)	0.857

AUC—area under the receiver operating characteristic curve; F1 Score—harmonic mean of precision and recall.

## Data Availability

The data presented in this study are available on request from the corresponding author due to ethical reasons.
